# Do green-blocking glasses enhance the nonvisual effects of white polychromatic light?

**DOI:** 10.1186/s40101-018-0189-3

**Published:** 2018-12-18

**Authors:** Soomin Lee, Naoshi Kakitsuba, Tetso Katsuura

**Affiliations:** 1 Center for Environment, Health and Sciences, Chiba University, Kashiwa, Japan; 2 Faculty of Science and Technology, Meijo University, Nagoya, Japan; 3 Graduate School of Engineering, Chiba University, Chiba, Japan

**Keywords:** Blue-blocking glasses, Green-blocking glasses, ipRGCs, Subadditive response, Nonvisual response

## Abstract

**Background:**

It is well known that light containing the blue component stimulates the intrinsically photosensitive retinal ganglion cells (ipRGCs) and plays a role in melatonin suppression and pupillary constriction. In our previous studies, we verified that simultaneous exposure to blue and green light resulted in less pupillary constriction than blue light exposure. Hence, we hypothesized that the nonvisual effects of polychromatic white light might be increased by blocking the green component. Therefore, we conducted an experiment using optical filters that blocked blue or green component and examined the nonvisual effects of these lights on pupillary constriction and electroencephalogram power spectra.

**Methods:**

Ten healthy young males participated in this study. The participant sat on a chair with his eyes facing an integrating sphere. After 10 min of light adaptation, the participant’s left eye was exposed to white pulsed light (1000 lx; pulse width 2.5 ms) every 10 s with a blue-blocking glasses, a green-blocking glasses, or control glasses (no lens), and pupillary constriction was measured. Then, after rest for 10 min, the participant was exposed a continuous white light of 1000 lx with a blue- or green-blocking glasses or control glasses and electroencephalogram was measured.

**Results:**

Pupillary constriction with the blue-blocking glasses was significantly less than that observed with the green-blocking glasses. Furthermore, pupillary constriction under the green-blocking glasses was significantly greater than that observed with the control glasses.

**Conclusions:**

A reduction in the green component of light facilitated pupillary constriction. Thus, the effects of polychromatic white light containing blue and green components on ipRGCs are apparently increased by removing the green component.

## Background

Humans adapted slowly for seven million years under sunlight, until the artificial light was invented. As modern technology has progressed, the artificial light has become unavoidable in a variety of situations. Accordingly, night-shift work and use of portable devices at nighttime have increased rapidly, such that humans are exposed to light, regardless of the time of day or night. Light that contains the blue component stimulates intrinsically photosensitive retinal ganglion cells (ipRGCs) and contributes to melatonin suppression [1, 2] and pupillary constriction [3–7]. Critically, blue light exposure in the evening induces sleep disturbances, transient eye discomfort, and headaches [8, 9]. In particular, it has been reported that the blue light emitted directly from computer displays suppresses melatonin secretion during the night [10, 11] and that the use of blue-blocking glasses inhibits this action [11–14]. Conversely, daytime blue light exposure has an acute preventive impact on light-induced melatonin suppression at night [15]. Furthermore, chronic daytime exposure to blue-enriched light has the potential to improve the subjective measures of alertness, concentration, and eye discomfort [9].

However, Figueiro et al. [16] found that simultaneous exposure to blue and green light resulted in reduced melatonin suppression, compared with monochromatic light exposure to blue or green light; this was referred to as a subadditive response to light. We also verified that simultaneous blue and green light exposure resulted in less pupillary constriction than blue light exposure [6, 7]. These findings indicated that the effect of blue light was inhibited by simultaneous exposure to green light. In addition, the melatonin suppression response to polychromatic white light was significantly lower than to monochromatic blue light [17, 18].

Hence, we hypothesized that the nonvisual effects of polychromatic white light might increase by blocking the green component. Therefore, we conducted a novel experiment by using optical filters that blocked the blue or green components and examined the nonvisual effects of light on pupillary constriction and electroencephalogram power spectra.

## Main text

### Study participants

Ten healthy young males (22 ± 0.5 years, 174.4 ± 3.6 cm, 63.2 ± 5.2 kg) with normal color vision participated in the experiment. Written informed consent was obtained from all subjects after a full explanation of the experimental purpose and protocol. This experiment was approved by the Ethics Committee of the Graduate School of Horticulture, Chiba University (#15-06).

### Lighting condition and transmittance of glasses

**Fig. 1:**
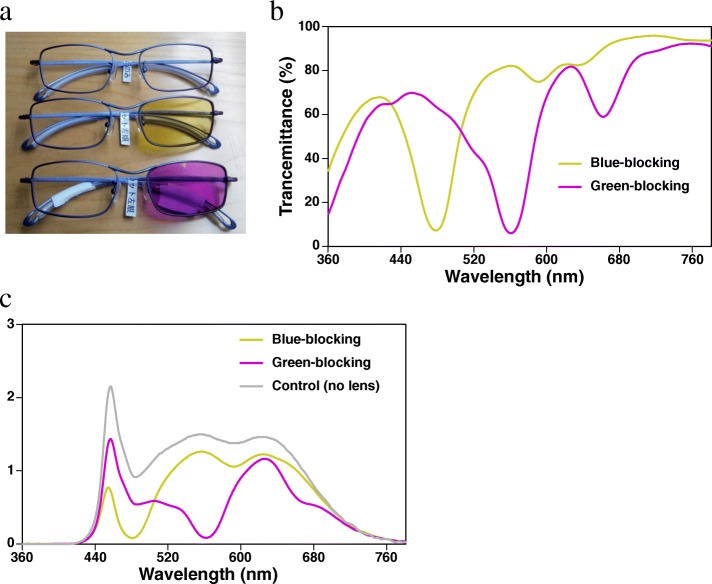
**a** The control glasses, blue-blocking glasses, and green-blocking glasses are shown in order from the top. **b** Spectral transmittance of blue-blocking glasses and green-blocking glasses. **c** Spectral irradiance of light of the source white LED light (no lens: control) and of the light through the blue-blocking and green-blocking glasses

We used white-pulsed LED light (1000 lx, pulse width 2.5 ms; 16 W24-AW2S, Kashinoki Sogyo Co., LTD.) for measuring pupillary constriction according to our previous studies [5–7] and white-continuous LED light (1000 lx) for electroencephalogram (EEG) measurement with white background light (100 lx), by using an integrating sphere (Takano Co., Nagano, Japan). We used a background light in order to saturate rods responses [19] and avoid the influence of rods on pupillary contraction as much as possible [7]. The illuminance of light was measured at each subject’s eye level by using a spectroradiometer (CL-500A, Konica Minolta Optics Co., Tokyo, Japan). The experiment was conducted in a climatic chamber in which the air temperature and relative humidity were set at 25 °C and 50%, respectively. Each participant wore blue-blocking glasses, green-blocking glasses, and control glasses (no lens) in each condition, respectively. These glasses contained only left-side lenses in order to measure the pupillary diameter (PD) of the right eye (Fig. 1a). Figure 1b, c show the spectral transmittance of these glasses and irradiance of the source white LED light and of the light through the blue-blocking and green-blocking glasses. Table 1 summarizes the relevant photometric measures of the source white LED light and of the light through each set of glasses [20].

**Table 1:** Characteristics of the light through each glasses conditions

Glasses condition	Blue-blocking	Green-blocking	Control (no lens)
Irradiance (μW/cm^2^)	240	184	345
Photon density (10^12^ photons/cm^2^/s)	716	537	988
Photon density (log photons/cm^2^/s)	14.9	14.7	15.0
Photopic illuminance (lx)	770	376	1008
Scotopic illuminance (lx)	1070	922	2025
Cyanopic lx (S-cone)	202	397	611
Melanopic lx (Melanopsin)	377	445	860
Rhodopic lx (Rod)	494	396	903
Chloropic lx (M-cone)	654	363	965
Erythropic lx (L-cone)	732	412	988

### Procedure and measurements

**Fig. 2:**
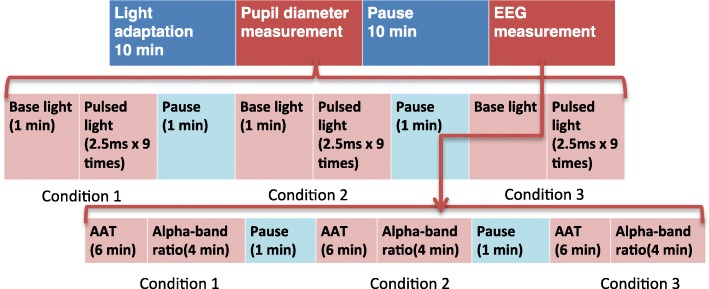
The experimental procedure

Each participant sat on a chair with his eyes facing the integrating sphere. Only his left eye was exposed to the light through the glasses, while his right eye faced the electronic pupillometer (FP-10000II, TMI, Tokyo, Japan). Each participant completed four steps of the experiment as follows: 10 min of background light exposure for light adaptation, pupil diameter measurement during pulsed light exposure, rest for 10 min, and EEG measurement at Fz, Cz, and Pz (Biopac Systems, CA, USA) under continuous LED light exposure. In the measurement of pupil diameter, each participant’s left eye was exposed nine times to the pulsed light every 10 s. This process was repeated three times for each of the three glasses conditions. Pupil diameter measurements were averaged for each subject and under each glasses condition. In the measurement of EEG, each participant underwent 6 min of Alpha Attenuation Test (AAT) and 4 min of EEG measurement to determine the alpha-band ratio (alpha-band power/(alpha-band power + beta-band power) × 100) under continuous LED light. This process was repeated three times for each of the three glasses conditions. Figure 2 shows a schematic of the experiment.

From the measurement of PD, we calculated the pupillary constriction ratio (% pupillary constriction) as follows: % pupillary constriction = [(baseline PD − minimum PD after light exposure)/baseline PD] × 100.

### Statistical analysis

We used one-way repeated measures analysis of variance (ANOVA) (SPSS Statistics Ver. 21, IBM, Armonk, NY, USA) to evaluate the effects of the glasses factor on pupillary constriction. Two-way repeated measures ANOVA (glasses factor × region factor) was conducted on EEG measurements. When significant effects were found, multiple comparisons of the glasses condition were performed by the Bonferroni method.

## Results

### Pupillary constriction

**Fig. 3:**
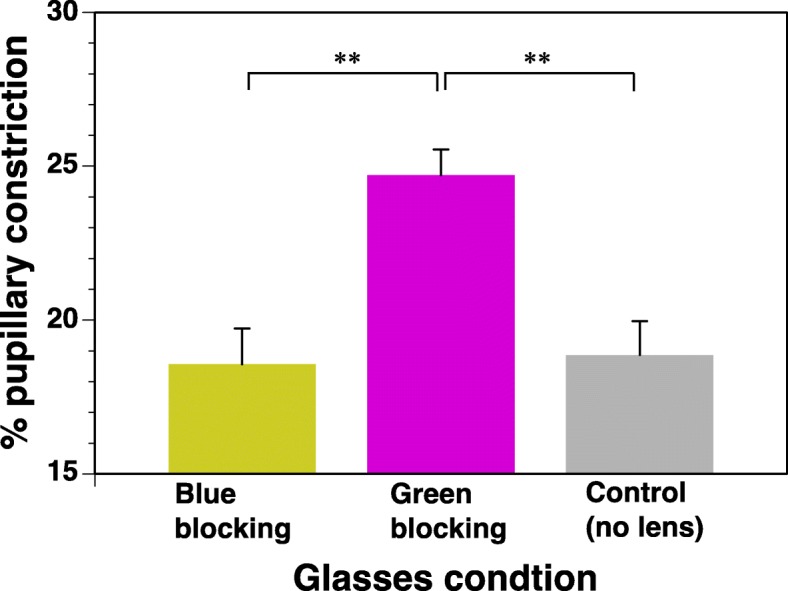
Pupillary constriction in three glasses conditions (means ± S.E., *n* = 10, **p < 0.01)

In the % pupillary constriction, there is significant difference among three glasses conditions [*F*(2, 34) = 15.6499, *p* = 0.001]. The % pupillary constriction in the blue-blocking glasses condition was significantly less than in the green-blocking condition. Furthermore, the % pupillary constriction in the green-blocking glasses condition, which contained the blue component of light but less green component, was significantly greater than the control glasses condition, which contained both blue and green components. There were no significant differences between the % pupillary constriction in the blue-blocking glasses condition and the control glasses condition (Fig. 3).

### Electroencephalogram

**Fig. 4:**
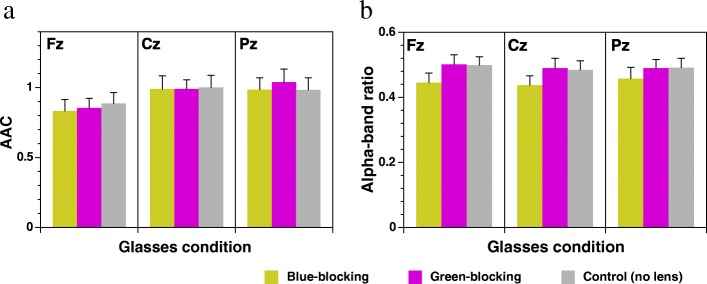
**a** Alpha Attenuation Test (AAT) and **b** alpha-band ratio in three glasses conditions at Fz, Cz, and Pz (means ± S.E., *n* = 10)

There were no significant differences in Alpha Attenuation Coefficient (AAC) [*F*(2, 18) = 0.2709 (Fz), *F*(2, 18) = 0.0135 (Cz), *F*(2, 18) = 0.48858 (Pz)] and alpha-band ratio [*F*(2, 18) = 2.1369 (Fz), *F*(2, 18) = 2.1058 (Cz), *F*(2, 18) = 1.2104 (Pz)] among the three glasses conditions (Fig. 4).

## Conclusions

In this study, we could not find any differences in AAC and alpha-band ratio among the three glasses conditions. We measured EEG during 10 min period for each glasses condition. It might be too short to make any differences in arousal level and EEG activity.

We found that the pupillary constriction in the blue-blocking glasses condition was less than in the green-blocking glasses condition. It might be inferred from the characteristics of ipRGCs that pupillary constriction in the blue-blocking glasses condition, which allowed minimal transmittance of the blue component of light, was markedly suppressed, compared with the green-blocking glasses condition, which allowed high transmittance of the blue component of light. Moreover, the most important finding was that pupillary constriction in the green-blocking glasses condition was significantly greater than in the control glasses condition, although the green-blocking glasses allowed approximately half the melanopic illuminance of the control glasses, as shown in Table 1.

It has been suggested that cone-derived color signals may influence nonvisual responses to light, such as pupillary light responses [21]. Woelders et al. [21] have demonstrated that M- and S-cones provide inhibitory input to the pupillary control system, whereas L-cones and melanopsin response present an excitatory role. These findings support a subadditive response to light, where the effects of blue light are reduced by green or polychromatic light exposure, as in the previous studies [6, 7] and the present study.

We also found that the pupillary constriction in the blue-blocking glasses and the control condition were almost same. We also hypothesized that the responses of ipRGCs might be reduced by inhibition from cones on simultaneous exposure to blue and green light in the control condition and might result in the same responses in the blue-blocking glasses condition, which had less blue component by nature.

Thus, the effects of polychromatic light, which contained blue and green components, on ipRGCs are apparently increased by removing the green component, as shown by using the green-blocking glasses in the present study. In addition, blue light exposure during the daytime improved nocturnal light-induced melatonin suppression [15]. If always wearing light-blocking lenses, adaptation to the changes in the spectral composition of light occurs [22]. However, such adapting effect does not occur in wearing glasses for several hours a day, and effective improvements can be expected [23]. Therefore, the use of green-blocking glasses during the daytime for several hours might improve these nonvisual effects.

In conclusion, the nonvisual effects of polychromatic white light were increased by blocking the green component of light. Therefore, we propose that the use of green-blocking glasses during the daytime for several hours might expand nonvisual effects (e.g., high arousal level) in the daytime and may improve nighttime sleep quality.

## Abbreviations

AAC: Alpha Attenuation Coefficient
AAT: Alpha Attenuation Test
ANOVA: Analysis of variance
EEG: Electroencephalogram
ipRGCs: Intrinsically photosensitive retinal ganglion cells
PD: Pupillary diameter
